# 6-Formyl Umbelliferone, a Furanocoumarin from *Angelica decursiv*a L., Inhibits Key Diabetes-Related Enzymes and Advanced Glycation End-Product Formation

**DOI:** 10.3390/molecules27175720

**Published:** 2022-09-05

**Authors:** Md Yousof Ali, Gerald W. Zamponi, Su Hui Seong, Hyun Ah Jung, Jae Sue Choi

**Affiliations:** 1 Department of Physiology and Pharmacology, Hotchkiss Brain Institute and Alberta Children’s Hospital Research Institute, Cumming School of Medicine, University of Calgary, Calgary, AB T2N 4N1, Canada; 2 Department of Food and Life Science, Pukyong National University, Busan 48513, Korea; 3 Division of Natural Products Research, Honam National Institute of Biological Resource, Mokpo 58762, Korea; 4 Department of Food Science and Human Nutrition, Jeonbuk National University, Jeonju 54896, Korea

**Keywords:** 6-FU, antidiabetic potentials, glucose uptake, molecular docking, advanced glycation end-products, kinetics

## Abstract

Over the years, great attention has been paid to coumarin derivatives, a set of versatile molecules that exhibit a wide variety of biological activities and have few toxic side effects. In this study, we investigated the antidiabetic potential of 6-formyl umbelliferone (6-FU), a novel furanocoumarin isolated from *Angelica decursiva*. Numerous pharmacological activities of 6-FU have been previously reported; however, the mechanism of its antidiabetic activity is unknown. Therefore, we examined the action of 6-FU on a few candidate-signaling molecules that may underlie its antidiabetic activity, including its inhibition of protein tyrosine phosphatase 1B (PTP1B), α-glucosidase, human recombinant aldose reductase (HRAR), and advanced glycation end-product (AGE) formation (IC_50_ = 1.13 ± 0.12, 58.36 ± 1.02, 5.11 ± 0.21, and 2.15 ± 0.13 μM, respectively). A kinetic study showed that 6-FU exhibited mixed-type inhibition against α-glucosidase and HRAR and competitive inhibition of PTP1B. Docking simulations of 6-FU demonstrated negative binding energies and close proximity to residues in the binding pockets of those enzymes. We also investigated the molecular mechanisms underlying 6-FU’s antidiabetic effects. 6-FU significantly increased glucose uptake and decreased PTP1B expression in insulin-resistant C2C12 skeletal muscle cells. Moreover, 6-FU (0.8–100 μM) remarkably inhibited the formation of fluorescent AGEs in glucose-fructose-induced human serum albumin glycation over the course of 4 weeks. The findings clearly indicate that 6-FU will be useful in the development of multiple target-oriented therapeutic modalities for the treatment of diabetes and diabetes-related complications.

## 1. Introduction

Diabetes mellitus (DM) is a metabolic disorder characterized by glucose intolerance and changes in lipid and protein metabolism. At present, more than 463 million people worldwide have diabetes, and that number could reach 574 million by 2030 and 700 million by 2045 [1]. According to the World Health Organization, diabetes will become the seventh leading cause of death by 2030 [2]. The proportion of people with DM is increasing rapidly worldwide, and therefore, the prevention and treatment of diabetes are urgent. Diabetes is divided into type 1 and type 2 (T2DM). Type 1 is a common, chronic, autoimmune disease that causes insulin dependence and is mostly diagnosed in young patients. T2DM is the most common form of diabetes, accounting for 90–95% of DM cases, and is characterized by insulin resistance and abnormal insulin secretion [3,4].

The enzymes that play a crucial role in T2DM are protein tyrosine phosphatase 1B (PTP1B), α-glucosidase, aldose reductase [5], and non-enzymatic glycation products called advanced glycation end-products (AGEs) [6]. Among them, the PTPs comprise more than a hundred biological enzymes, and PTP1B is understood to modulate the insulin signaling pathway [5]. PTP1B is widely expressed in insulin-sensitive peripheral tissues such as adipose and skeletal muscle tissues, and it can cause both insulin resistance and T2DM [7,8]. PTP1B is a key negative regulator of the leptin and insulin signaling pathways responsible for energy expenditure, glucose homeostasis, and body weight control [9]. Additionally, PTP1B can directly bind to the activated insulin receptor and insulin receptor substrate-1 to dephosphorylate phosphotyrosine residues and thereby control insulin action [8], decrease translocation of the insulin-sensitive glucose transporter type [4,10], and regulate leptin signaling pathways [11]. Thus, PTP1B is an attractive therapeutic target for T2DM.

α-glucosidase is a crucial enzyme affecting the digestion and absorption of different carbohydrates, particularly starch [12]. α-glucosidase hydrolyzes starch into dextrin and oligosaccharides into glucose [13]. When it is transported to blood vessels, it increases the postprandial blood glucose level and causes diabetes [12,14]. Therefore, reducing postprandial hyperglycemia and slowing glucose absorption by inhibiting α-glucosidase is another therapeutic approach to restoring insulin sensitivity. Currently, α-glucosidase has been identified as the main target enzyme in preventing and treating T2DM.

Aldose reductase (AR, EC 1.1.1.21) is a key aldo-keto reductase enzyme of the polyol pathway that catalyzes glucose to sorbitol, along with the parallel conversion of NADPH to NADP^+^ [15]. Furthermore, sorbitol is converted to fructose by the presence of the sorbitol dehydrogenase enzyme, and that occurs in an NAD^+^-dependent manner [16]. AR is known to play key roles in the pathogenesis of diabetes-related complications such as neuropathy, nephropathy, and retinopathy [17]. AR inhibitors, which are critical for managing factors involved in the onset and progression of DM, are also being developed [16]. Therefore, AR is an attractive target for researchers who are designing and developing drugs to treat and manage long-term diabetic complications.

AGEs are a heterogeneous group of compounds generated via a non-enzymatic browning reaction called the Maillard reaction [18]. Generally, AGEs are generated by nonenzymatic reactions between reducing sugars such as glucose and fructose and free amino groups of proteins, lipids, and nucleic acids [19]. More than twenty AGEs, including Nε-(carboxyethyl) lysine, Nε-(carboxymethyl) lysine, pyrraline, and pentosidine, are considered potentially harmful to humans [20]. Humans are exposed to AGEs in two ways, via endogenous AGEs generated from abnormal glucose metabolism or lipid peroxidation or via exogenous AGEs from various foods. Exogenous AGEs can easily enter the bloodstream and accumulate in the body [20]. Many studies have suggested that the buildup of AGE levels that are induced by long-term hyperglycemia causes DM-related complications such as retinopathy, nephropathy, and cardiomyopathy [6,21,22]. Therefore, inhibiting AGE formation or AGE cross-linking is widely recognized as an effective way to treat diabetic complications.

A rare hydroxycoumarin derivative in nature, 6-formyl umbelliferone (6-FU) has been isolated from *Angelica decursiva*, a perennial herb spread on grasslands, hillsides, and sparse forests within Korea, China, and Japan. *A. decursiva* is commonly used in traditional Korean medicine as an analgesic, antipyretic, and antitussive [23]. The leaves of *A. decursiva* are also used as a salad green in Korea and are confirmed to be nontoxic [24]. Moreover, this plant has been used as a traditional Chinese medicine for asthma, thick phlegm, and upper respiratory tract infections [23,25,26]. A wide range of biological activities has been reported for this valuable herb due to its high content of diverse coumarin derivatives, including psoralen, hydroxycoumarin, dihydroxanthyletin, dihydropyran, and dihydropsoralen [23]. Our laboratory previously isolated various coumarin derivatives from *A. decursiva* that have potential antidiabetic [27,28,29,30], anti-Alzheimer [30,31,32,33], antioxidant, anti-inflammatory [26,34], and antihypertensive activities [35]. We recently reported the neuroprotective [36], anti-Alzheimer [32], and anti-inflammatory [37] activity of 6-FU. Caffieri et al. [38] also reported the mitochondrial effects of a novel apoptogenic molecule of 6-FU. No other biological studies of 6-FU have been reported.

Therefore, to the best of our knowledge, we are the first to screen the antidiabetic properties of 6-FU. In this work, we evaluated the underlying mechanisms of the inhibitory potential of 6-FU by using enzyme assays against PTP1B, α-glucosidase, human recombinant AR (HRAR), and AGEs. We also explore the mode of inhibition and molecular interactions between 6-FU and those enzymes. Additionally, we examined the glucose uptake stimulatory effects of 6-FU and the expression of PTP1B in insulin-resistant C2C12 cells.

## 2. Results and Discussion

### 2.1. Inhibitory Activity of 6-FU against PTP1B, α-Glucosidase, HRAR, and AGE Formation

To evaluate its anti-DM potential, 6-FU was tested in vitro using PTP1B, α-glucosidase, HRAR, and AGE formation inhibitory assays (Table 1). The 6-FU exhibited strong inhibitory activity against PTP1B, α-glucosidase, and HRAR, with IC_50_ values of 1.13 ± 0.12, 58.36 ± 1.02, and 5.11 ± 0.21 µM, respectively. For comparison, the values for the positive controls used in those respective assays were ursolic acid (IC_50_ value of 4.28 ± 0.32 µM), acarbose (IC_50_ value of 123.88 ± 0.87 µM), and quercetin (IC_50_ value of 3.14 ± 0.17 µM). Additionally, 6-FU showed promising AGE inhibitory activity, with an IC_50_ value of 2.15 ± 0.13 µM, compared with the positive control (aminoguanidine), which had an IC_50_ value of 527.43 ± 4.55 µM. Our laboratory previously reported antidiabetic activity of various coumarin derivatives, including psoralen, hydroxycoumarin, dihydroxanthyletin, dihydropyran, and dihydropsoralen from *A. decursiva* by inhibiting various enzyme systems [27,28,29,30]. However, in this study, we investigated the antidiabetic activity of a rare 6-FU furanocoumarin that exhibited potential inhibitory activities of various enzymes that are responsible for diabetes and diabetes-related complications. When compared to previously reported coumarin derivatives, 6-FU demonstrated strong inhibitory activities against PTP1B, and α-glucosidase.

### 2.2. Kinetic Parameters of 6-FU against PTP1B, α-Glucosidase, and HRAR

**Figure 1:**
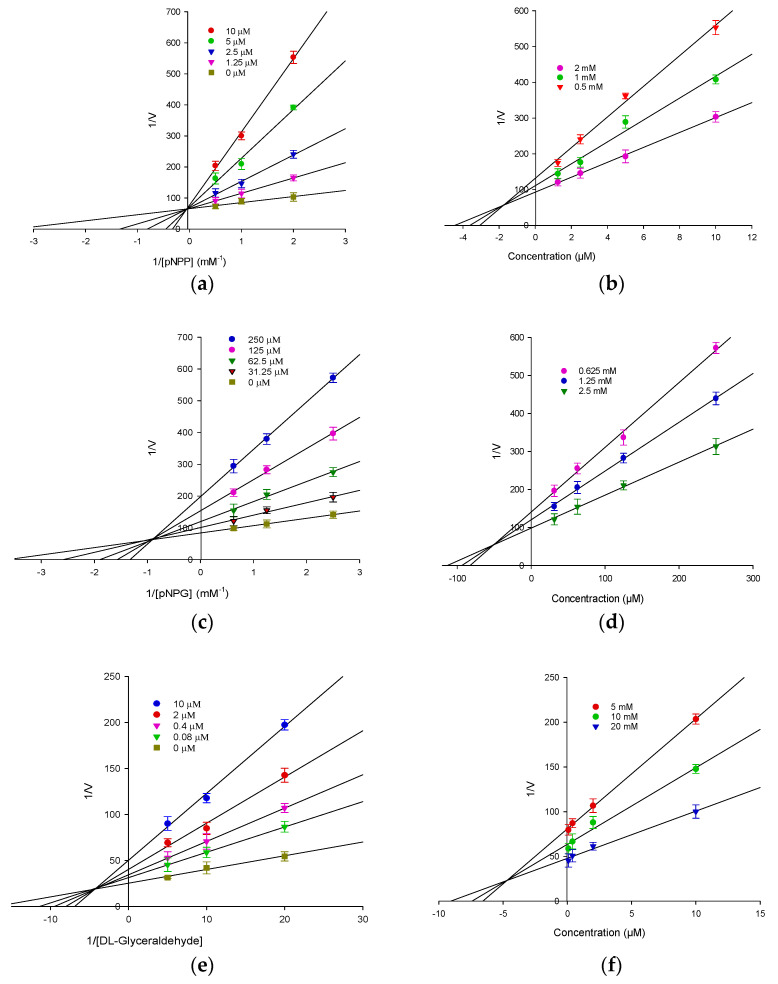
Lineweaver-Burk and Dixon plots for protein tyrosine phosphatase 1B (PTP1B), α-glucosidase and human recombinant aldose reductase (HRAR) inhibition by 6-FU. (**a**) Lineweaver-Burk plot for PTP1B inhibition by 6-FU was analyzed in the presence of different concentrations of sample as follows: 0 µM (■), 1.25 µM (▼), 2.5 µM (▼), 5 µM (●), and 10 µM (●). (**b**) Dixon plots of PTP1B inhibition by 6-FU: 2 mM (●), 1.0 mM (●), and 0.5 mM (▼) for pNPP. (**c**) Lineweaver-Burk plot for α-glucosidase inhibition by 6-FU was analyzed in the presence of different concentrations of sample as follows: 0 µM (■), 31.25 µM (▼), 62.5 µM (▼), 125 µM (●), and 250 µM (●). (**d**) Dixon plots of α-glucosidase inhibition by 6-FU: 2.5 mM (▼), 1.25 mM (●), and 0.625 mM (●) for pNPG. (**e**) Lineweaver-Burk plot for HRAR inhibition by 6-FU was analyzed in the presence of different concentration of sample as follows: 0 µM (■), 0.08 µM (▼), 0.4 µM (▼), 2.0 µM (●), and 10 µM (●). (**f**) Dixon plots of HRAR inhibition by 6-FU: 5 mM (●), 10 mM (●), and 20 mM (▼) for _DL-_glyceraldehyde.

To further explore the enzymatic inhibition by 6-FU, kinetic analyses were performed at different concentrations of the corresponding substrate (pNPP for PTP1B, pNPG for α-glucosidase, and _DL_-glyceraldehyde for HRAR) and 6-FU. Figure 1 and Table 1 show the results of the enzymatic kinetic analysis of 6-FU. As shown in Figure 1a,b, 6-FU showed competitive PTP1B inhibition, with a K_i_ value of 1.72 μM. It showed mixed type inhibition for α-glucosidase, with a K_i_ value of 49.52 μM (Figure 1c,d). As shown in Table 1, 6-FU also showed mixed-type inhibition for HRAR, with a K_i_ value of 4.87 µM (Figure 1e,f). Because the K_i_ value represents the optimal concentration at which an enzyme–inhibitor complex forms, this value plays an important role in the development of preventive and therapeutic agents.

### 2.3. Molecular Docking Analysis of 6-FU Interactions with PTP1B

**Figure 2:**
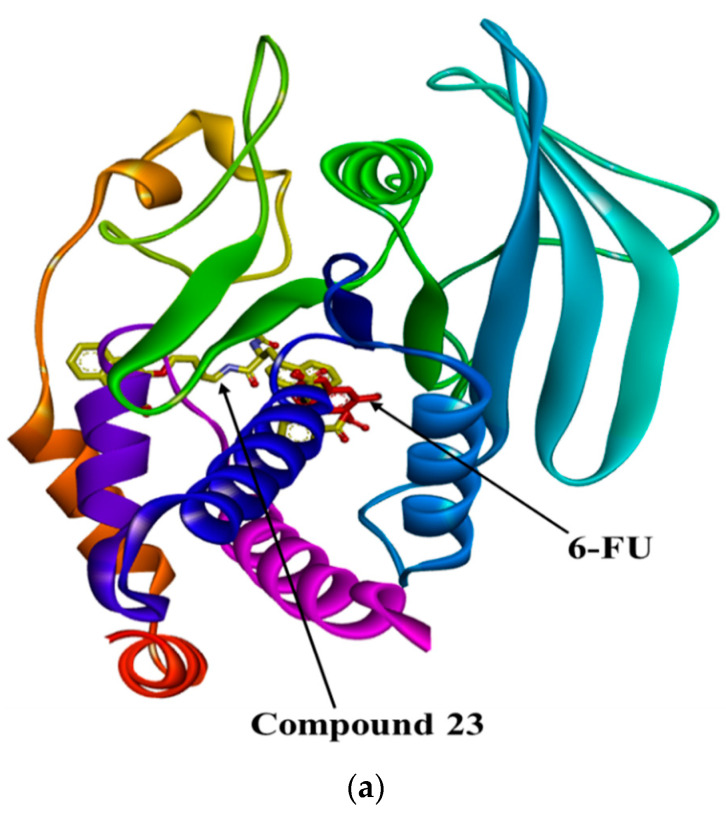

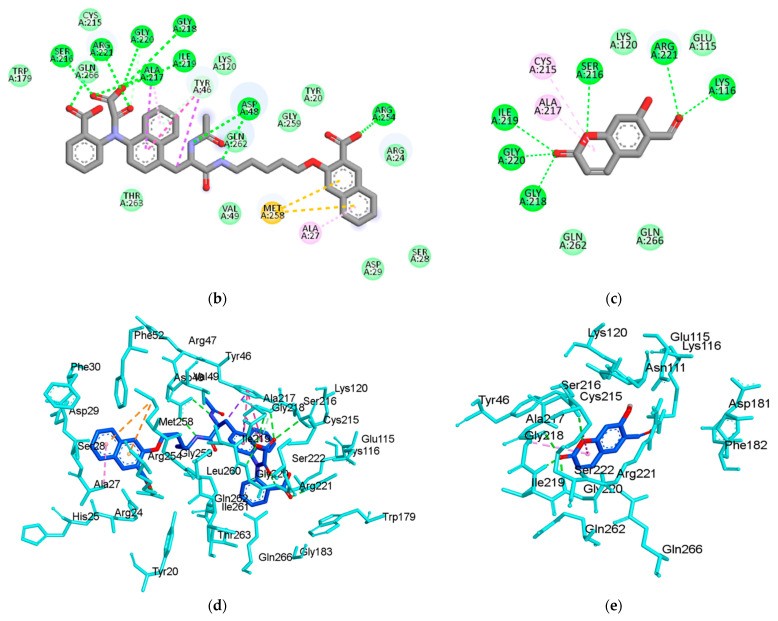
Molecular docking model for protein tyrosine phosphatase 1B (PTP1B) inhibition by 6-FU. (**a**) Overlapping of the docked 6-FU (red) and compound **23** (yellow) within the active site of PTP1B (PDB: 1NNY). 2D interactions of compound **23** (**b**) and 6-FU (**c**) inside the active pocket of PTP1B. Molecular docking model for PTP1B inhibition by 6-FU and diagrams of 3D ligand interactions and major binding interactions of inhibitors with the 1NNY active site: compound **23** (**d**), and 6-FU (**e**). The interactions are represented in green (conventional hydrogen bonding), pink (π-alkyl interactions), gold accent (π-sulfur), purple (π-sigma), dark pink (π-π T shaped), and light green (π-donor).

A molecular docking simulation in Autodock Vina was used to investigate the binding position of 6-FU within the active site of PTP1B (Figure 2a). The molecular modeling of the cognate ligand, 3-({5-[(*N*-acetyl-3-{4-[(carboxycarbonyl)(2-carboxyphenyl)amino]-1-naphthyl}-l-alanyl)amino]pentyl}oxy)-2-naphthoic acid (compound **23**) of PTP1B showed several important interactions, including hydrogen bonding with Asp48, Arg254, Arg221, Ser216, Gly220, Gly218, Ile219, and Ala217 within the active pocket of PTP1B (Figure 2b). Moreover, π-alkyl interactions were observed between compound **23** and Ala27 and Ala217. The other interactions exhibited by compound **23** were π-sulfur with Met258, π-sigma with Tyr46 and Ala217, π-π stacked with Tyr46, a carbon hydrogen bond with carbon hydrogen bond, and a π-donor-hydrogen bond with Gly220 (Table 2). Next, our selected compound, 6-FU, was docked within the active pocket of PTP1B, and it exhibited mostly conventional hydrogen bonding with Arg221, Gly218, Gly220, Ile219, Lys116, and Ser216 (Figure 2c). Furthermore, it showed π-alkyl interactions with Ala217 and Cys215. The crystalline structure of PTP1B consists of one catalytic loop that contains the catalytic site with the Cys215 residue and a P loop that consists of His214, Ser216, Ala217, Gly218, Ile219, Gly220, and Arg221 [39]. The 3D interactions between compound **23** and 6-FU are shown in Figure 2d,e. 6-FU exhibited multiple hydrogen and π-alkyl bond interactions within the active pocket of PTP1B, which could be the reason for its stabilization inside the protein and enzymatic inhibition. Additionally, Figure 2a suggests that 6-FU was well accommodated inside the active pocket and that the presence of 6-FU inside the catalytic cavity allowed the protein to make conformational changes. In parallel with the in vitro enzymatic inhibition, 6-FU demonstrated good docking results, with interactions like those of the co-crystalline ligand, and good binding energies. These findings thus complement the in vitro results.

### 2.4. Molecular Docking Analysis of 6-FU Interactions with α-Glucosidase

**Figure 3:**
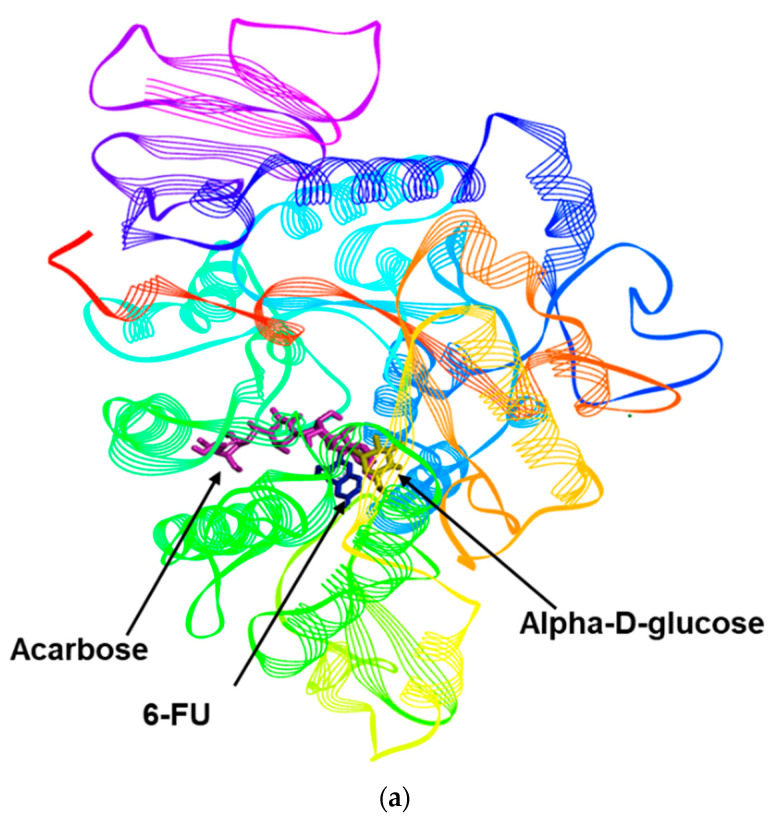

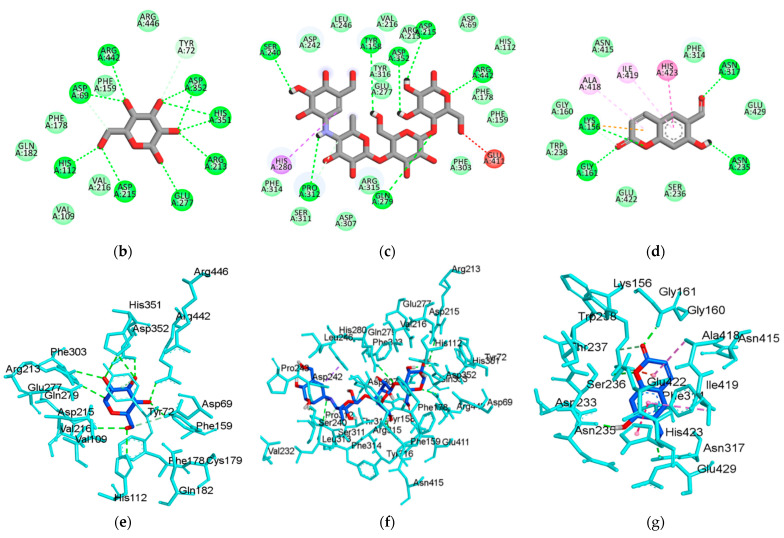
Molecular docking model for α-glucosidase inhibition by 6-formyl umbelliferone (6-FU). (**a**) Overlapping of the docked acarbose (dark pink), alpha D-glucose (yellow), and 6-FU (blue) within the active site of α-glucosidase (PDB: 3A4A). 2D interactions of alpha D-glucose (**b**), acarbose (**c**), and 6-FU (**d**) inside the active pocket of α-glucosidase. Molecular docking model for α-glucosidase inhibition by 6-FU and diagrams of 3D ligand interactions and major binding interactions between the inhibitors and the active site of 3A4A: alpha D-glucose (**e**), acarbose (**f**), and 6-FU (**g**). The interactions are represented in green (conventional hydrogen bonding), pink (π-alkyl interactions), gold accent (π-cation), purple (π-sigma), dark pink (π-π T shaped), red (donor-donor acceptor), and light green (carbon hydrogen bond).

In the molecular docking simulation of 6-FU with α-glucosidase, the Autodock Vina docking program showed that ligand–enzyme complexes between 6-FU or acarbose and alpha-D-glucose were stably posed in the same pocket (Figure 3a). Upon docking, the most likely binding mode of the co-crystalline ligand of 3A4A (alpha-D-glucose) exhibited several important interactions, including hydrogen bonding with Asp69, Arg442, Arg213, Asp352, Asp215, Glu277, His112, and His351 within the α-glucosidase active pocket (Figure 3b and Table 2). However, Tyr72 and Asp69 were found to exhibit carbon hydrogen bonds with alpha-D-glucose (Figure 3b). Moreover, when the positive control (acarbose) used in the bioassay was docked within the α-glucosidase active pocket, it demonstrated largely hydrogen bonding with Asp352, Asp215, Arg442, Gln279, Pro312, Ser240, and Tyr158 with varying bond lengths (Figure 3c). In addition to hydrogen bonds, acarbose exhibited carbon hydrogen bonds, π-sigma, and unfavorable accepter-accepter interactions with Pro312, His280, and Glu411, respectively, within 3A4A active pocket (Figure 3c). As shown as Figure 3d and Table 2, 6-FU formed conventional hydrogen bonds with Asn235, Asn317, Gly161, and Lys156; π-alkyl interactions with Ala418 and Ile419; a π-π T-shaped interaction with His423; and π-cation and carbon hydrogen bond interactions with Lys156. The 3D interactions of alpha-D-glucose, acarbose, and 6-FU are shown in Figure 3e–g.

### 2.5. Molecular Docking Analysis of 6-FU Interactions with HRAR

**Figure 4:**
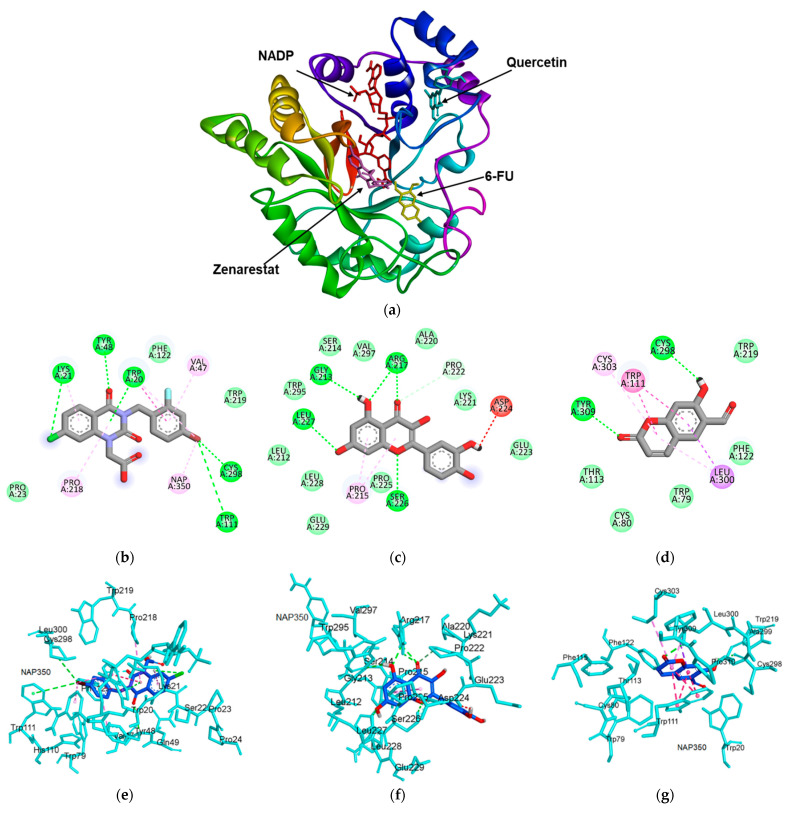
Molecular docking model for human recombinant aldose reductase (HRAR) inhibition by 6-formyl umbelliferone (6-FU). (**a**) Overlapping of the docked 6-FU (yellow), zenarestat (dark pink), NADP (red), and quercetin (cyan) within the active site of HRAR (PDB: 1IEI). 2D interactions of zenarestat (**b**), quercetin (**c**), and 6-FU (**d**) inside the active pocket of HRAR. Molecular docking model for HRAR inhibition by 6-FU and diagrams of 3D ligand interactions and major binding interactions between the inhibitors and the active site of 1IEI: zenarestat (**e**), quercetin (**f**), and 6-FU (**g**). The interactions are represented in green (conventional hydrogen bonding), pink (π-alkyl interactions), purple (π-sigma), dark pink (π-π T shaped), and red (donor-donor acceptor).

The molecular docking simulation for HRAR showed that the ligand–enzyme complexes of 6-FU, quercetin, and zenarestat were stably posed in the same pocket (Figure 4a). Upon docking, the probable binding mode of the co-crystalline ligand of 1IEI (zenarestat) exhibited several important interactions, including hydrogen bonds with Cys298, Lys21, Tyr48, Trp111, and Trp20; π-alkyl interactions with Trp20, Pro218, Nap350, and Lys21; an alkyl interaction with Val47; and a π-π stacked interaction with Trp20 (Figure 4b and Table 2). The 7-chloro-2,4-dioxoquinazolin-1-yl part of zenarestat was inclined toward the cofactor, NAP350, whereas the 4-bromo-2-fluorophenyl moiety of zenarestat was found to align near the hydrophilic pocket. The positive control (quercetin) used in the bioassay also docked within the HRAR active pocket and showed largely hydrogen bonding with Arg217, Gly213, Leu227, and Ser226 with varying bond lengths (Figure 4c and Table 2). In addition to hydrogen bonds, quercetin had π-alkyl, unfavorable donor-donor, and carbon hydrogen bond interactions with Pro215, Asp224, and Pro222, respectively, within the active pocket of 1IEI. As shown as Figure 4d and Table 2, the key interactions between 6-FU and the binding site of HRAR were π-π stacked interactions with Trp111, π-alkyl with Cys303 and Leu300, and π-sigma with Leu300. Additionally, hydrogen bonds with Cys298 and Tyr309 were revealed near the active pocket of the enzyme. The 3D interactions of zenarestat, quercetin, and 6-FU are shown in Figure 4e–g.

### 2.6. Molecular Docking Analysis of 6-FU Interactions with HAS

**Figure 5:**
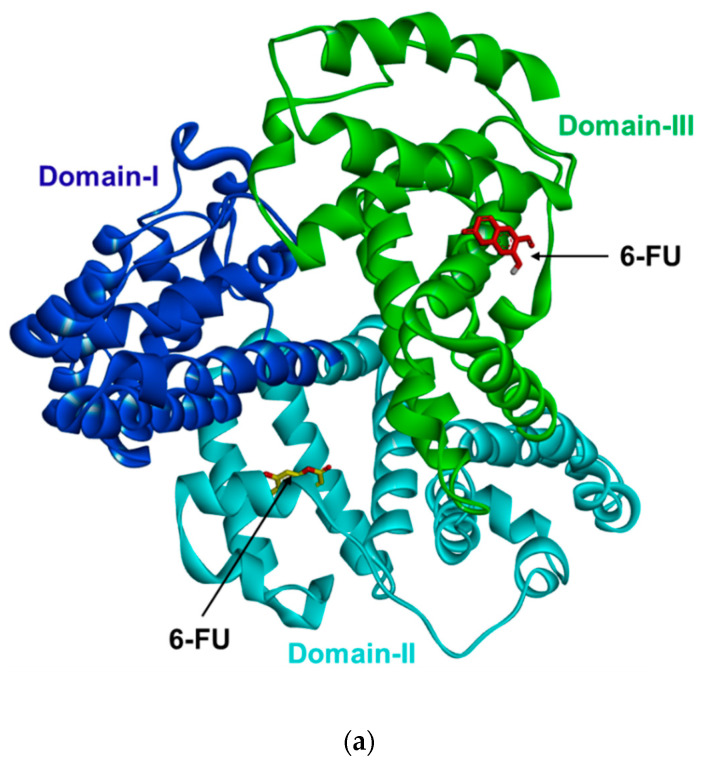

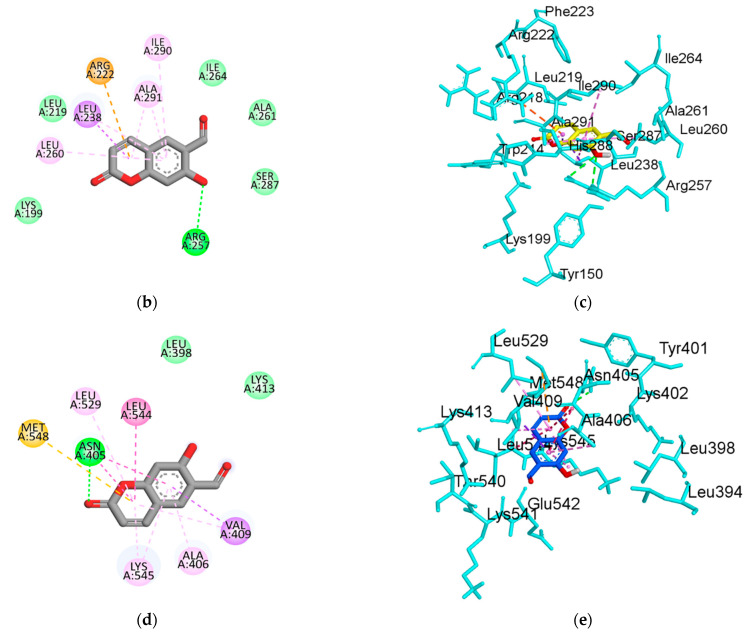
Molecular docking model for human serum albumin (HSA) inhibition by 6-formyl um-belliferone (6-FU). (**a**) Overlapping of the docked 6-FU (yellow) in subdomain IIA (binding site I) of HSA and 6-FU (red) in subdomain IIIA (binding site II) of HSA (PDB: 1AO6). The HSA domains are colored blue for domain I, cyan for domain II, and green for domain III. 2D interactions of 6-FU inside subdomain IIA of HSA (**b**) and 3D interactions and major binding interactions between the inhibitors and subdomain IIA of HSA (**c**). 2D interactions of 6-FU inside subdomain IIIA of HSA (**d**) and 3D interactions and major binding interactions between the inhibitors and subdomain IIIA of HSA (**e**). The interactions are represented in green (conventional hydrogen bonding), pink (π-alkyl interactions), orange (π-cation), purple (π-sigma), dark pink (amide-π stacked), and gold accent (π-sulfur).

The X-ray crystalline structure of HSA (PDB: 1AO6) consists of three main domains, domain I (residues 1–195), subdomain-IIA (also called site-I, residues 196–383), and subdomain-IIIA (also called site-II, residues 384–585) [40,41]. Molecular docking between 6-FU and HSA showed that 6-FU binds to both site-I and site-II (Figure 5a). As illustrated in Table 2, site-I and site-II of 6-FU exhibited −6.9 and −6.6 kcal/mol binding affinity to HSA, respectively, indicating that 6-FU was more inclined to bind at site-I. As shown in Figure 5b,c, 6-FU binding involved the formation of a key hydrogen bond between the Arg257 residue of HSA and the 6-FU hydroxyl group. In addition, site-I of 6-FU exhibited π-alkyl interactions with Leu260, Ala291, Leu238, and Ile290; a π-sigma interaction with Leu238; and a π-cation interaction with Arg222. As shown in Figure 5d,e, 6-FU binding to site-II involved the formation of a hydrogen bond between the Asn405 residue of HSA and the 6-FU ketone group, along with a π-sulfur bond with Met548 and π-alkyl interactions with Ala406, Leu529, Lys545, and Val406. Other interactions of 6-FU within binding site-II of HSA were π-sigma interactions with Val409, an amide-π stacked interaction with Leu544, and an amide-π stacked interaction with Asn403. In general, Arg and Lys are the major glycation sites in the protein, and the properties of the neighboring amino acids contribute greatly to its glycation level [42]. Other studies also found that Ile, Leu, Phe, and Arg strongly enhanced the reactivity of Lys in different Lys- containing compounds [42,43]. In this study, the Ile, Leu, Lys, and Arg involved in the interactions between 6-FU and HSA might alleviate the glycosylation activity of neighboring potential glycation sites. It was previously reported that various small molecules, including urolithin A [41], chrysin, and luteolin [42], formed hydrogen bonds with Arg257 and had hydrophobic interactions with Arg 222, Leu238, Leu260, Ala291, Ile290, Lys199, Lys545, Ala406, Leu544, Leu529, Ala406, and Val409 in HSA, which enhanced the stability of HSA and prevented glycation and AGE formation. In this study, 6-FU showed similar binding residues, which could improve the stability of HSA and prevent glycation and AGE formation.

### 2.7. Effect of 6-FU on Glucose Uptake and PTP1B Expression in Insulin-Resistant C2C12 Skeletal Muscle Cells

**Figure 6:**
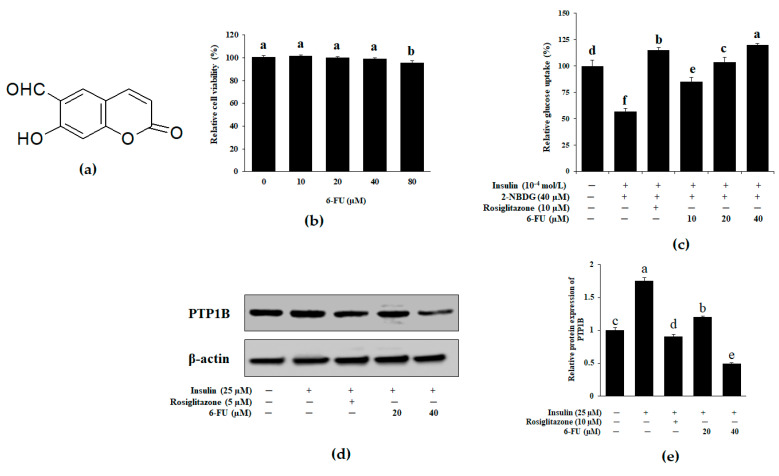
(**a**) Chemical structure of 6-FU. (**b**) Effect of 6-FU on C2C12 cell viability. Cell viabilities were assessed using the 3-(4,5-dimethylthiazol-2-yl)-2,5-diphenyl tetrazolium bromide (MTT) assay. (**c**) Effect of 6-FU on insulin-stimulated glucose uptake in insulin-resistant C2C12 skeletal muscle cells. Glucose uptake was assayed using the fluorescent D-glucose analogue 2-[N-(7-nitrobenz-2-oxa-1, 3-diazol-4-yl) amino]-2-deoxy-D-glucose (2-NBDG), and 100 nM insulin was used to induce insulin resistance. Cells were treated for 24 h with different concentrations of 6-FU and rosiglitazone, and then the insulin-stimulated 2-NBDG uptakes were measured. The results are expressed as the means ± SEMs of three separate experiments. ^a–f^ Different letters indicate statistical differences among the means of each rosiglitazone and 6-FU concentration. (**d**) Effect of 6-FU on protein tyrosine phosphatase 1B (PTP1B) levels in insulin-resistant C2C12 cells. C2C12 muscle cells were treated with the indicated concentrations of 6-FU for 12 h or 100 nM insulin for 60 min in serum-free Dulbecco’s modified Eagle medium (DMEM). Then, 2-NBDG was added at a final concentration of 20 µM in glucose-free DMEM for 30 min. (**e**) The relative densities of PTP1B versus β-actin and protein band intensities were quantified by densitometry. Results were normalized to β-actin levels and are presented as the means ± SEMs of three separate experiments. ^a–f^ Different letters indicate significant differences among the means of each rosiglitazone and 6-FU concentration. ^c^ Indicates normal C2C12 cells, ^a^ Indicates insulin resistant C2C12 cells. Results were analyzed by ANOVA and Duncan’s test (*p* < 0.05).

Insulin resistance is a fundamental feature of T2DM, and muscle cells are significant locations of energy consumption, storage, and balance as an insulin target [44]. Skeletal muscle is the core target organ in the human body that consumes glucose and plays a vital role in the pathogenesis of T2DM [45]. It is also one of the main tissues in the human body, constituting approximately 50% of its volume, and is known to be important in postprandial glucose homeostasis [46]. It has been reported that skeletal muscle cell metabolism is seriously decreased in diabetic patients [45,47]. Additionally, several studies have reported that natural compounds enhance glucose metabolism in muscle cells by increasing glucose uptake [48,49,50]. Therefore, understanding insulin resistance in skeletal muscle could be an effective strategy for developing drugs to treat diabetes. The chemical structure of 6-FU is shown in Figure 6a. Here, we examined 6-FU’s cytotoxicity and ability to increase glucose uptake in C2C12 cells (Figure 6). After incubation for 24 h, C2C12 cells were treated with 6-FU at concentrations up to 80 μM. As shown in Figure 6b, 6-FU was not cytotoxic up to 40 μM, so concentrations <40 μM were used in the subsequent glucose uptake assay. To assess the ability of 6-FU to increase glucose uptake, the 2-NBDG uptake assay was performed in insulin-resistant C2C12 cells. The 5 μM rosiglitazone positive control significantly increased insulin-stimulated glucose uptake in insulin-resistant C2C12 cells. 6-FU also significantly enhanced insulin-stimulated 2-NBDG uptake by insulin-resistant C2C12 cells at concentrations of 10, 20, and 40 μM, compared with the control (Figure 6c). These results indicate that 6-FU is involved in glucose uptake signaling pathways in muscle cells. PTP1B negatively regulates insulin signaling and increases in its activity and expression are implicated in the pathogenesis of insulin resistance [9,51]. Therefore, PTP1B inhibitors are potential therapeutic candidates to restore insulin sensitivity and treat T2DM. In this study, PTP1B expression in insulin-resistant C2C12 cells was affected by exposure to selected concentrations of 6-FU for 24 h (Figure 6d). As shown in Figure 6e, treating insulin-resistant C2C12 cells with 40 μM 6-FU decreased the PTP1B expression level.

### 2.8. Effect of 6-FU on Fluorescent AGE Formation

**Figure 7:**
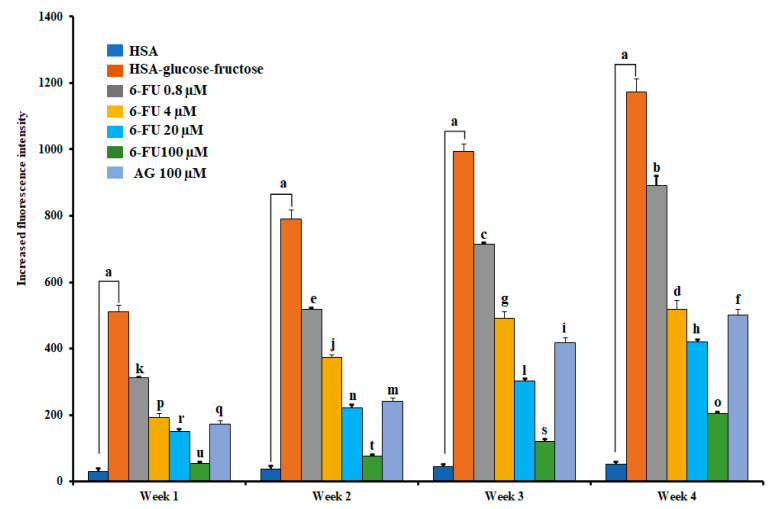
The inhibitory effect of 6-formyl umbelliferone (6-FU) and aminoguanidine (AG) on the formation of fluorescent advanced glycation end-products (AGEs) in the human serum albumin (HSA)–glucose–fructose system. Data are expressed as the mean ± SEM from a minimum of duplicate independent experiments. ^a^ Represents a significant increase compared with the HSA values. Significant differences within each group are denoted by letters. ^a–u^ Means with different letters differed significantly in the ANOVA and Duncan’s test analysis (*p* < 0.05).

AGEs are a chemically heterogeneous group of compounds formed by the Maillard reaction when reducing sugars react nonenzymatically with amine residues, proteins, lipids, and nucleic acids [18]. It has been reported that the accumulation of AGEs is triggered by protein alterations in various tissues that change their biochemical and physiochemical properties [21]. Moreover, AGE overproduction plays a major role in various disorders, including diabetic complications [18], Alzheimer’s disease, connective tissue diseases [18], vascular inflammation, cell apoptosis [52], and end-stage renal disease [53]. Therefore, the possibility of consuming antiglycation agents to improve or prevent diabetic complications has attracted interest. Generally, glucose and fructose are the most common reducing sugars found in human blood. Although glucose plays a crucial role in AGE formation, fructose undergoes protein glycation that is much faster than glucose [5,6]. Therefore, we monitored AGE formation weekly by measuring the fluorescence intensity of HSA–glucose–fructose solutions. We found a significant increase in HSA glycation and fluorescence with increasing incubation time, as shown in Figure 7. When 6-FU was added to the reaction media containing the HSA–glucose–fructose system, a significant reduction in fluorescence intensity was observed. At week 4 of incubation, the percentage of inhibition of AGE formation was 24.04%, 55.73%, 64.18%, and 82.60% at 6-FU concentrations of 0.8, 4.0, 20, and 100 μM, respectively. The positive control (aminoguanidine) inhibited AGE formation by 57.23% at a concentration of 100 μM. Previously, several studies have reported [41,42,43] that late-stage glycation products in the HSA-glucose, HSA-glyoxal, or HSA-ovalbumin system were inhibited by numerous compounds, but those results showed lower AGE inhibition than 6-FU.

## 3. Materials and Methods

### 3.1. Chemicals and Reagents

Yeast α-glucosidase, acarbose, *p*-nitrophenyl phosphate (*p*NPP), *p*-nitrophenyl α-D-glucopyranoside (*p*NPG), quercetin (>95% purity), ursolic acid (>90% purity), acarbose (>95% purity), rosiglitazone (>98% purity), insulin from bovine pancreas, phenylmethylsulfonyl fluoride (PMSF), bovine serum albumin, human serum albumin (HSA), dimethyl sulfoxide (DMSO), 3-(4,5-dimethylthiazol-2-yl)-2,5-diphenyl tetrazolium bromide (MTT), guanidine hydrochloride, 2,4-dinitrophenylhydrazine, nitroblue tetrazolium, 5,5′-dithiobis (2-nitrobenzoic acid), thioflavin T, 1-deoxy-1-demorpholino-D-fructose, L-cysteine, DL-glyceraldehyde dimer, D-(–)-fructose, D-(+)-glucose, and aminoguanidine hydrochloride were purchased from Sigma-Aldrich Co. (St. Louis, MO, USA). PTP1B (human recombinant) was purchased from Biomol^®^ International LP (Plymouth Meeting, PA, USA). Dulbecco’s modified Eagle medium (DMEM), penicillin–streptomycin (P/S), 0.25% trypsin-ethylenediaminetetraacetic acid (EDTA), fetal bovine serum (FBS), sodium pyruvate, and nonessential amino acids were purchased from Gibco-BRL Life Technologies (Grand Island, NY, USA). Dithiothreitol (DTT) was purchased from Bio-Rad Laboratories (Hercules, CA, USA). The fluorescent D-glucose analog and glucose tracer 2-[N-(7-nitrobenz-2-oxa-1, 3-diazol-4-yl) amino]-2-deoxy-D-glucose (2-NBDG) was purchased from Life Technologies (Carlsbad, CA, USA). Sodium azide was purchased from Junsei Chemical Co. (Tokyo, Japan). HRAR (0.4 units) was purchased from Wako Chemicals (Osaka, Japan). PTP1B, β-actin, and secondary antibodies were obtained from Santa Cruz Biotechnology (Dallas, TX, USA). All other chemicals and solvents used were purchased from E. Merck, Fluka, or Sigma–Aldrich (St. Louis, MO, USA), unless otherwise stated.

### 3.2. Isolation of 6-FU from A. decursiva

The whole plant powder of *A. decursiva* was refluxed with 99.8% methanol (MeOH) for 3 h (3 × 10 L). The total filtrate was then concentrated until dry in vacuo at 40 °C to render the MeOH extract (389.58 g). This extract was suspended in distilled water and then successively partitioned with dichloromethane (CH_2_Cl_2_), ethyl acetate (EtOAc), and *n*-butanol (*n*-BuOH) to yield CH_2_Cl_2_, EtOAc, *n*-BuOH, and H2O fractions. The EtOAc fraction was also subjected to silica gel column chromatography using CH_2_Cl_2_–MeOH (10:1→0:1, gradient) to get 20 subfractions (F-1 to F-20). Repeated chromatography of F-6 over a silica gel column using CH_2_Cl_2_–MeOH (20:1→0:1, gradient) yielded 6-FU (30 mg). The structure of the compounds was confirmed by ^1^H- and ^13^C-NMR spectroscopy and comparison with published data [32].

### 3.3. Assay for PTP1B Inhibition

The PTP1B inhibitory activity was evaluated using *p*NPP according to the modified procedure reported by Ali et al. [27]. Ursolic acid was used as the positive control.

### 3.4. α-Glucosidase Inhibitory Assay

The enzyme inhibition study was performed spectrophotometrically using the procedure reported by Ali et al. [27]. Acarbose dissolved in 10% DMSO was used as the positive control.

### 3.5. HRAR Inhibition Assay

HRAR inhibition was examined as described by Ali et al. [28]. Briefly, AR activity was determined by measuring the decrease in NADPH absorption at 340 nm over a period of 1 min on an Ultrospec^®^2100pro UV/Visible spectrophotometer (Cytiva, Buckinghamshire, UK). SWIFT II Applications software (Amersham Biosciences, NJ, USA) was used for all data analyses. Quercetin and zenarestat well-known AR inhibitors were used as the positive control. The inhibition percentage (%) was calculated as described in the RLAR assay, except that the DA sample per min value represented the reduction in absorbance for 1 min with the test samples and substrate. The ability of each sample to inhibit HRAR was expressed as an IC_50_ value (µM), which was calculated from the log-dose inhibition curve.

### 3.6. In Vitro Glycation of HSA

The formation of glycated HSA-fructose-glucose was examined according to a previously published method [6]. To prepare the AGE reaction solution, 20 mg/mL HSA in 50 mM sodium phosphate buffer (pH 7.4) was added to 0.2M fructose, 0.2 M glucose, and 0.02% sodium azide to prevent bacterial growth. The reaction mixture (3.8 mL) was then mixed with 200 μL of various concentrations of 6-FU and aminoguanidine hydrochloride dissolved in 10% DMSO. The control was prepared using HSA and sugars, and a blank was prepared using only HSA in the same buffer. The reaction mixtures were incubated at 37 °C for 4 weeks. Aliquots of the reaction mixtures were then assayed to determine the presence of AGEs.

### 3.7. Inhibition of AGE Formation

The ability of the isolates to inhibit AGE formation was evaluated according to a previously published method with slight modifications [6]. To prepare the AGE reaction solution, 20 mg/mL HSA in 50mM sodium phosphate buffer (pH 7.4) was added to 0.2M fructose and 0.2 M glucose, along with 0.02% sodium azide to prevent bacterial growth. The reaction mixture (950 μL) was then combined with various concentrations of 6-FU (50 μL, test concentration ranging from 0.8 to 100 μM) dissolved in 10% DMSO. After incubating the mixture at 37 °C, AGE formation in the aliquots was determined at weekly intervals using a spectrofluorometric detector (FLx800 microplate fluorescence reader, Bio-Tek Instrument, Inc., Winooski, VT, USA) with excitation and emission wavelengths of 350 nm and 450 nm, respectively. The nucleophilic hydrazine compound aminoguanidine was used as a reference in the AGE assay.

### 3.8. Determination of the Kinetic Parameters of PTP1B, α-Glucosidase, and HRAR Inhibition via Lineweaver-Burk and Dixon Plots

To determine the inhibition mechanism, enzymatic inhibition at various concentrations of 6-FU was evaluated by monitoring the effects of different concentrations of substrates via Dixon plots. Dixon plots for PTP1B inhibition by 6-FU were obtained in the presence of 0.5, 1, and 2 mM *p*NPP. The test concentrations of 6-FU in the PTP1B kinetic analysis were 0, 1.25, 2.5, 5.0, and 10 µM. Dixon plots for α-glucosidase inhibition by 6-FU were performed in the presence of 0.625, 1.25, and 2.5 mM *p*NPG. The test concentrations of 6-FU used in the α-glucosidase kinetic analysis were 0, 31.25, 62.5, 125, and 250 µM. Both enzymatic procedures followed the PTP1B and α-glucosidase assay methods. Dixon plots for HRAR inhibition by 6-FU were performed in the presence of 5, 10, and 20 mM of DL-glyceraldehyde as the substrate. The test concentrations of 6-FU used in the HRAR kinetic analysis were 0, 0.08, 0.4, 2, and 10 µM. The Dixon plot is a graphical method [1/enzyme velocity (1/*V*) against inhibitor concentration (I)] for determining the type of enzyme inhibition and was used to determine the dissociation or inhibition constant (K*_i_*) for the enzyme–inhibitor complex. Thus, the type of enzyme inhibition was determined by interpreting the Lineweaver–Burk plots, and the inhibition constants (K*_i_*) were determined by interpreting the Dixon plots.

### 3.9. Molecular Docking Studies

The crystal structures of the targeted proteins were downloaded from the Protein Data Bank (pdb) before we conducted the docking analysis. To investigate the binding poses of 6-FU inside the active receptor pockets, the protein structures for PTP1B, (PDB ID: 1NNY) [39], α-glucosidase (PDB ID: 3A4A) [54], HRAR (PDB ID: 1IEI) [55], and HSA (PDB 1D: 1AO6) [56] were downloaded from pdb. These protein structures were regulated using an X-ray diffraction method. The reported heteroatom compounds were removed, and the protein was regarded as ligand free. Water molecules were also removed from the protein structure for the docking simulation using Accelrys Discovery Studio 4.1 (DS 4.1) (http://www.accelrys.com, accessed on 1 September 2022; Accelrys, Inc. San Diego, CA, USA). Polar hydrogen atoms were added to the protein using an automated docking tool, Autodock 4.2.6.[57,58]. The docking studies for the 6-FU, quercetin, acarbose, and co-crystalline ligands were performed without modifying the default parameters. For the docking studies, the binding areas of compound **23**, alpha-D-glucose, and zenarestat were considered the most affirmative regions for docking with the proteins. The 2D structures of 6-FU were drawn with MarvinSketch (www.chemaxon.com, accessed date 1 July 2022; Chemaxon, Life Sciences, Informatics, Cheminformatics, Budapest, Hungary) and converted into 3D pdb format using DS 4.1. Autodock Vina is an open-source program for simulating molecular docking that offers significantly better average accuracy in its binding mode predictions than Autodock 4 [59]. Autodock Vina improves the speed and accuracy of docking with a new scoring function, efficient optimization, and multithreading. A grid box sized 60 × 60 × 60 with a spacing of 1.0 Å between the grid points was used to cover almost all the favorable protein binding sites. The X, Y, Z centers were: PTP1B (31.15, 28.65, and 21.49), HRAR (0.14, −0.04, and −0.6), α-glucosidase (21.28, −0.75, and 18.63), and HSA (29.52, 31.81, and 23.49). In the docking studies, the selected ligand (6-FU) was examined to find qualified binding poses for each compound. The binding aspects of the PTP1B, α-glucosidase, HRAR, and HSA residues and their corresponding binding affinity scores are regarded as the best molecular interactions. The results are reported and were analyzed using Discovery Studio Visualizer (Accelrys Software Inc. 2013, San Diego, CA, USA). Two-dimensional figures of the compound–protein interactions were created using Discovery Studio Visualizer (Accelrys Software Inc. 2013). All docking simulations were performed using an Intel^®^ Core ™ i7-4510 CPU @ 4.00GHz with Windows 10 and a 64-bit operating system as the molecular docking simulation environment.

### 3.10. Cell Culture and Cell Viability Assay

The C2C12 cell line (skeletal muscle cells) was purchased from the American Type Culture Collection (Manassas, VA, USA). The C2C12 cells were cultured at 37 °C in DMEM supplemented with 10% FBS and P/S (Hyclone, Mordialloc, VIC, Australia) in a humidified atmosphere with 5% CO_2_. The tetrazolium dye colorimetric test (MTT) was used to determine cell viability. First, the C2C12 cells were cultured in 96-well plates (2 × 10^5^ cells/well) for 24 h. After they reached 90% confluence, they were treated with various concentrations of 6-FU. After 24 h of incubation, the MTT reagent was added to each well, and the plate was incubated for 2 h at 37 °C. The media were removed, and the wells were washed twice with PBS (pH 7.4). To measure the proportion of surviving cells, the medium was replaced with 100 μL DMSO (100%). The resulting absorbance values were measured at 570 nm with a microplate reader (Molecular Devices, Sunnyvale, CA, USA).

### 3.11. Muscle Cell Differentiation and Glucose Uptake Assay

The C2C12 cells were cultured in 96-well plates (2 × 10^5^ cells/well) with DMEM containing 10% FBS and 1% P/S at 37 °C and exposed to a 5% CO_2_ atmosphere. When the cells reached confluence, they were subjected to differentiation in DMEM supplemented with 2% horse serum for 5 days. Then the cells were starved in low-glucose serum-free DMEM for 24 h. Subsequently, the 2-NBDG assay was performed to evaluate glucose uptake [5]. The cells were treated with various concentrations of 6-FU and insulin (100 nM) for 12 h, followed by 20 μM 2-NBDG for 24 h. To stop the response, the cells were washed three times with ice-cold PBS, and then the 2-NBDG fluorescence intensity was measured on a microplate reader (FL × 800, Bio-Tek Instruments Inc., Winooski, VT, USA) at 490 nm (excitation) and 535 nm (emission). Five replicate wells were established, and each experiment was replicated three times. Rosiglitazone was used as the positive control.

### 3.12. Preparation of Protein Lysates and Western Blot Analysis

Insulin-resistant C2C12 cells (2 × 10^5^ cells/well) in 6-well plates were treated with different concentrations of 6-FU for 24 h. After stimulation with 100 nM insulin for 30 min at 37 °C, the cells were washed three times with ice-cold PBS, collected, and lysed with sample buffer (50 mM HEPES, pH 7.5, 150 mM NaCl, 2.5 mM EDTA, 0.5% NP40, 1 mM PMSF, 1 mM DTT, 0.2% aprotinin, 0.5% leupeptin, 20 mM NaF, and 1 mM Na3VO4) on ice. After incubation for 30 min, the insoluble materials were removed by centrifugation at 25,000× *g* for 20 min. The protein concentrations were determined using a modified Bradford protein assay kit. Total protein (50 μg) was subjected to dodecyl sulfate–polyacrylamide gel electrophoresis and transferred to a polyvinylidene difluoride membrane. The membranes were blocked in blocking buffer (5% skim milk in Tris-buffered saline containing 0.1% Tween 20 (TBST)) and incubated with primary antibodies overnight at 4 °C, followed by incubation with appropriate secondary antibodies for 4 h at room temperature. The membranes were washed three times with TBST for 40 min. The bands were detected using Super Signal West Pico chemiluminescence substrate (Pierce, Rockford, IL, USA) according to the manufacturer’s instructions. The immunoreactive protein bands were detected using an Odyssey scanning system (LI-COR, Lincoln, NE, USA). A densitometric analysis of the data was performed using a cooled CCD camera system (EZ-Capture II) and CS Analyzer version 3.00 software (ATTO Co., Tokyo, Japan).

### 3.13. Statistical Analysis

All results are expressed as the mean ± SEM of triplicate samples. Results were analyzed using one-way ANOVA and Student’s t-testing where appropriate (Systat Inc., Evaston, IL, USA). Values of * *p*< 0.05, ** *p*< 0.001, and *** *p*< 0.0001 were considered statistically significant.

## 4. Conclusions

In summary, 6-FU shows promising antidiabetic and antidiabetic complication potential by inhibiting PTP1B, α-glucosidase, HRAR, and AGE formation and improving insulin sensitivity. The evidence shows that 6-FU from *A. decursiva* enhances insulin-sensitivity by downregulating PTP1B expression and promoting the uptake of glucose in insulin-resistant C2C12 muscle cells. Moreover, 6-FU effectively inhibited the formation of fluorescent AGEs and regulated the AR-related polyol pathway, which are important therapeutic targets for the treatment of DM. Based on these findings, *A. decursiva*–derived 6-FU coumarin might be effective in controlling hyperglycemia by inhibiting α-glucosidase and attenuating the insulin signaling pathway via PTP1B inhibition. It might also ameliorate DM-associated complications by inhibiting AR- and AGE-related pathways. Further in vivo and cellular-based signaling pathway tests are needed to clarify the detailed mechanisms of coumarin in the brain and other organs.

## Figures and Tables

**Table 1:** Inhibitory effects and enzyme kinetic analysis of 6-FU on protein tyrosine phosphatase 1B (PTP1B), α-glucosidase, human recombinant aldose reductase (HRAR), and advanced glycation end-product (AGE).

		IC_50_ (µM) ^a^		
Test Sample	PTP1B	α-Glucosidase	HRAR	AGE
6-FU	1.13 ± 0.12 ***	58.36 ± 1.02 ***	5.11 ± 0.21 **	2.15 ± 0.13 ***
K_i_^b^	1.72	49.52	4.87	
Inhibition type ^c^	Competitive	Mixed	Mixed	
Ursolic acid ^d^	4.28 ± 0.32 **			
Acarbose ^e^		123.88 ± 0.87 **		
Quercetin ^f^			3.14 ± 0.17 **	
Aminoguanidine ^g^				527.43 ± 4.55 *
Zenarestat ^h^			0.63 ± 0.01 ***	

^a^ The 50% inhibition concentrations (IC_50_, µM) are expressed as the mean ± SEM of triplicates. ^b^ Inhibition constants (K_i_) were determined using Dixon plots. ^c^ Inhibition type was determined by interpreting the Dixon plot and Lineweaver-Burk plot. ^d–h^ Positive controls. *p< 0.05, ** *p* < 0.001, and *** *p* < 0.0001 indicate significance differences from control.

**Table 2:** Binding energies and binding interactions of 6-FU, cognate ligands, and positive controls in protein tyrosine phosphatase 1B (PTP1B), α-glucosidase, human recombinant aldose reductase (HRAR), and human serum albumin (HSA) using the Autodock vina docking program and visualization by Discovery Studio Visualizer.

Target Enzymes	PDB ID	Ligands	Binding Energies (Kcal/mol)	Hydrogen Bonds, Interacting Residues, and Bonding Distance	Hydrophobic Interactions
Protein tyrosine phosphatase 1B (PTP1B)	1NNY	6-formyl umbelliferone (6-FU)	−8.1	Gly218 (2.89 Å), Gly220 (1.97Å), Ile219 (2.10 Å), Ser216 (2.75 Å), Arg221 (2.58 Å), Lys116 (2.29 Å)	Ala217 (π-alkyl, 4.50 Å), Cys215 (π-alkyl, 4.84 Å)
		Compound **23**	−8.6	Asp48 (2.82 and 2.64 Å), Arg254 (2.86 Å), Arg221(2.80 and 3.05 Å), Ser216 (3.14 Å), Gly220 (2.72 Å), Gly218 (3.39 Å), Ile219 (3.01 Å), Ala217 (2.69 Å)	Ala27 (π-alkyl, 5.09 Å), Ala217 (π-alkyl, 5.36 Å), Met258 (π-sulfur, 5.59 and 5.91 Å), Tyr46 (π-sigma, 3.57 Å), Ala217 (π-sigma, 3.87 Å), Tyr46 (π-π stacked 5.21 Å), Tyr46 (carbon hydrogen bond, 3.94 Å), Gly220 (π-donor-hydrogen bond, 3.88 Å)
α-Glucosidase	3A4A	6-formyl umbelliferone (6-FU)	−7.9	Asn235 (1.83 Å), Asn317 (2.17 Å), Gly161 (2.05 Å), Lys156 (3.08 Å)	Ala418 (π-alkyl, 4.69 Å), Ile419 (π-alkyl, 5.24 Å), His423 (π-π T-shaped 5.10 Å), Lys156 (π-cation, 4.01 Å), Lys156 (carbon hydrogen bond, 3.38 Å)
		Acarbose	−8.2	Asp352 (2.38 Å), Asp215 (2.90 Å), Arg442 (2.31 Å), Gln279 (3.02 Å), Pro312 (3.08 Å), Ser240 (2.90 Å), Tyr158 (2.73 Å)	Pro312 (carbon hydrogen bond, 2.68 Å), His280 (π-sigma, 3.93 Å), Glu411 (unfavorable accepter-accepter, 2.93 Å)
		Alpha-D-glucose	−6.8	Asp69 (2.63 Å), Arg442 (2.78 Å), Arg213 (2.89 Å), Asp352 (2.67 and 2.52Å), Asp (2.88 Å), Glu277 (2.75 Å), His112 (2.77 Å), His351 (3.01 and 3.01 Å)	Tyr72 (π-donor hydrogen bond 3.93 Å), Asp69 (carbon hydrogen bond, 3.41 Å)
Human recombinant aldose reductase (HRAR)	1IEI	6-formyl umbelliferone (6-FU)	−7.8	Cys298 (2.19 Å), Tyr309 (2.10 Å)	Leu300 (π-sigma, 3.64 Å), Leu300 (π-alkyl, 4.48 Å), Cys303 (π-alkyl, 5.02 Å), Trp111 (π-π stacked, 4.57 and 4.21 Å)
		Zenarestat	−8.0	Cys298 (2.45 Å), Lys21 (2.29 Å), Tyr48 (2.91 Å), Trp111(2.98 Å), Trp20 (2.78 Å)	Trp20 (π-alkyl, 4.83 Å), Pro218 (π-alkyl, 4.83 Å), Lys21 (π-alkyl, 5.23 Å), Val47 (alkyl, 4.48 Å), Nap350 (π-alkyl, 4.44 Å), Trp20 (π-π stacked, 5.56 Å)
		Quercetin	−8.2	Arg217 (2.32 and 4.09 Å), Gly213 (2.59 Å), Leu227 (2.64 Å), Ser226 (2.66 Å)	Pro215 (π-alkyl, 5.07 and 3.99 Å), Asp224 (unfavorable donor-donor, 3.68 Å), Pro222 (carbon hydrogen bond, 3.49 Å)
Human serum albumin (HSA)	1AO6	6-formyl umbelliferone (Site-I)	−6.9	Arg257 (3.46 Å)	Leu260 (π-alkyl, 5.29 Å), Ala291(π-alkyl, 4.64 and 4.28 Å), Leu238 (π-alkyl, 5.31Å), Ile290 (π-alkyl, 5.26 Å), Leu238 (π-sigma, 4.77 Å), Arg222 (π-cation, 4.32 Å)
		6-formyl umbelliferone (Site-II)	−6.6	Asn405 (2.38 Å)	Ala406 (π-alkyl, 4.84 Å), Leu529 (π-alkyl, 4.95 Å), Lys545 (π-alkyl, 4.08 and 3.98 Å), Val406 (π-alkyl, 5.17 Å), Met548 (π-sulfur, 5.71 Å), Val409 (π-sigma, 3.91 Å), Leu544 (amide-π stacked, 4.33 Å), Asn403 (amide-π stacked, 4.54 and 4.59 Å)

## Data Availability

All data are available within the article.

## Sample Availability

Samples of the compounds are available from the authors.
